# Immune checkpoint inhibitors-induced thyroid dysfunction improves the prognosis of patients with lung cancer: a meta-analysis and systematic review

**DOI:** 10.3389/fendo.2025.1743245

**Published:** 2026-01-20

**Authors:** Yang Dong, Yuxia Li, Xingqiao Peng, Wei Fang

**Affiliations:** 1 Department of Respiratory Medicine, Nan’an District People’s Hospital, Chongqing, China; 2 Department of Pediatrics, Shenzhen Shenshan People’s Hospital, Shenzhen, China; 3 Department of Oncology, Army Medical Center of PLA, Chongqing, China

**Keywords:** immune checkpoint inhibitor, lung cancer, meta-analysis, prognosis, systematic review, thyroid dysfunction

## Abstract

**Background:**

Several studies have explored the impact of immune checkpoint inhibitor (ICI)–induced immune-related thyroid dysfunction on the prognosis of patients with lung cancer. However, inconsistencies remain among the results of different studies. Therefore, we conducted a meta-analysis to evaluate the impact of immune-related thyroid dysfunction on the prognosis of lung cancer, aiming to provide evidence-based support for clinical treatment.

**Methods:**

We searched PubMed, Embase, the China National Knowledge Infrastructure (CNKI), and the Cochrane Library to identify studies on the association between immune-related thyroid dysfunction and the prognosis of patients with lung cancer. The search period spanned from the establishment of each database to November 2025. Two researchers independently performed literature screening, data extraction, and assessment of the risk of bias in the included studies. A meta-analysis was performed using RevMan 5.3 software.

**Results:**

A total of 14 studies involving 2,252 patients with lung cancer were included. The meta-analysis showed that ICI-induced immune-related thyroid dysfunction improved the overall survival (OS) (HR = 0.47, 95% confidence interval, CI [0.39, 0.56], *P* < 0.00001) and progression-free survival (PFS) (HR = 0.44, 95% CI [0.38, 0.52], *P* < 0.00001) in patients with lung cancer. Subgroup analysis revealed that in both Asian and non-Asian populations, ICI-induced immune-related thyroid dysfunction was associated with improved OS (Asian: HR = 0.53, 95% CI [0.43, 0.66], *P* < 0.00001; non-Asian: HR = 0.32, 95% CI [0.22, 0.45], *P* < 0.00001) and PFS (Asian: HR = 0.45, 95% CI [0.38, 0.55], *P* < 0.00001; non-Asian: HR = 0.42, 95% CI [0.30, 0.58], *P* < 0.00001) in patients with lung cancer. Additionally, both ICI-induced hypothyroidism and hyperthyroidism improved OS in patients with lung cancer (hypothyroidism: HR = 0.47, 95% CI [0.33, 0.68], *P* < 0.00001; hyperthyroidism: HR = 0.28, 95% CI [0.15, 0.53], *P* < 0.00001).

**Conclusions:**

In patients with lung cancer receiving immunotherapy, the development of thyroid dysfunction (a treatment-related adverse event) may indicate a more robust immune response to the therapy, which is associated with improved treatment outcomes. This finding provides evidence-based support for predicting the prognosis of patients with lung cancer treated with ICIs.

**Systematic review registration:**

https://www.crd.york.ac.uk/prospero/, identifier CRD420251267541.

## Introduction

Lung cancer is one of the most common cancers worldwide in terms of both incidence and mortality, and it is also a leading cause of cancer-related death (1, 2). It is classified into non-small cell lung cancer (NSCLC) and small-cell lung cancer (SCLC) (3). Among these, NSCLC accounts for approximately 80%–85% of cases, mainly including subtypes such as adenocarcinoma, squamous cell carcinoma, and large cell carcinoma (4–6). The etiology and pathogenesis of lung cancer involve smoking, environmental exposure, genetic susceptibility, and complex molecular biological mechanisms (3, 7–9). Treatment options for lung cancer primarily include surgical resection, chemotherapy, radiotherapy, targeted therapy, and immunotherapy (6, 10). However, most patients with lung cancer are diagnosed at an advanced stage, thus missing the optimal opportunity for surgical resection (11). The treatment for advanced lung cancer mainly relies on radiotherapy and chemotherapy, but the treatment outcome is still unsatisfactory, with an overall 5-year survival rate of approximately 21.7% (12). Therefore, it is urgent to explore alternative treatment options to improve patient outcomes.

In recent years, the emergence of immune checkpoint inhibitors (ICIs) has brought a revolutionary breakthrough in lung cancer treatment, significantly improving the survival prognosis of patients and becoming a key approach in the field of lung cancer treatment (10). Multiple studies have shown that ICIs significantly improve the survival rates of patients with NSCLC, melanoma, renal cell carcinoma, and other types of cancer (13–16).

ICIs enhance the body’s antitumor immune response by blocking inhibitory receptors on immune cells, including programmed death receptor 1 (PD-1), programmed cell death-ligand 1 (PD-L1), and cytotoxic T-lymphocyte–associated antigen 4 (CTLA-4), among others (17). They have been widely applied in the treatment of advanced solid tumors, significantly improving patients’ clinical benefits, prolonging survival, and maintaining a favorable safety profile (18). However, immune-related adverse events (irAEs) may occur during ICI treatment. IrAEs refer to inflammatory toxic reactions induced during ICI treatment, which are usually associated with excessive activation of the immune system and can affect multiple organ systems, including the digestive tract, endocrine glands, skin, liver, heart, and lung, among others (19, 20). These events may lead to immune system overactivation, endocrine disorders, cutaneous adverse reactions, and neurological complications, among others. Among them, immune-related thyroid dysfunction is the most common endocrine adverse event in ICI treatment, including hyperthyroidism, hypothyroidism, and painless thyroiditis, (21).

Given the critical role of thyroid function in regulating systemic physiology, any thyroid dysfunction during lung cancer treatment may not only affect patients’ quality of life but also potentially impact treatment outcomes and prognosis. Some studies suggested an association between immune-related thyroid dysfunction and treatment response as well as survival outcomes in patients with lung cancer (22, 23). However, conclusions from different studies are inconsistent: some indicate that patients with lung cancer who develop immune-related thyroid dysfunction may have better treatment responses and survival outcomes (24–26), while others fail to identify a clear correlation (27–29). Therefore, we conducted this meta-analysis to evaluate the impact of immune-related thyroid dysfunction on the prognosis of patients with lung cancer (including indicators such as overall survival (OS) and progression-free survival (PFS), to more accurately clarify the relationship between the two and provide more reliable evidence-based medical support for clinical treatment.

## Materials and methods

This meta-analysis followed the Preferred Reporting Items for Systematic Reviews and Meta-Analyses (PRISMA) 2020 guidelines (30–32) (**Supplementary Table S1**). The protocol was registered in PROSPERO (CRD420251267541).

### Inclusion

Patients (P): Patients with lung cancer.

Exposure (E): Development of immune-related thyroid dysfunction in patients with lung cancer.

Control (C): No development of immune-related thyroid dysfunction in patients with lung cancer.

Outcomes (O): Progression-free survival (PFS) or overall survival (OS) data that could be directly extracted or calculated.

Study design (S): Cohort study or case-control study.

### Exclusion criteria

1. Literature with duplicated data.

2. Reviews, case reports, comments, commentaries, letters, conference abstracts, and other non-original research literature.

3. Literature with no access to original data.

### Search strategy

We searched PubMed, Embase, the China National Knowledge Infrastructure (CNKI), and the Cochrane Library for relevant literature on the impact of immune-related thyroid dysfunction on the prognosis of patients with lung cancer, covering the period from the establishment of each database to November 2025. There was no restriction on the language of the literature. The search was performed using MeSH terms combined with free words. Boolean logic “OR” was used to combine terms within each group, and “AND” was used to combine the result sets to identify studies focusing on both topics. Search terms were as follows: ICI, programmed cell death 1 inhibitor, programmed death ligand 1 inhibitor, PD-1 inhibitor, PD-L1 inhibitor, immunotherapy, thyroid dysfunction, hypothyroidism, hyperthyroidism, thyrotoxicosis, immune-related adverse event, irAE, prognosis, survival, OS, PFS, lung cancer, lung neoplasm, pulmonary cancer, pulmonary neoplasm, and NSCLC, among others. The detailed search strategy can be found in the **Supplementary Materials** (**Supplementary Table S2**).

### Study selection and data extraction

References retrieved from the aforementioned databases were imported into EndNote software to remove duplicates. Then, two researchers independently screened the titles and abstracts of the remaining studies, excluding those that clearly did not meet the inclusion criteria, to complete the preliminary selection. The full texts of the shortlisted studies were reviewed individually, and the two researchers jointly determined the final included studies and cross-checked the results. Data extracted from the included studies included publication year, country of origin of the study participants, type of lung cancer, type of ICIs, source of survival data, type of thyroid dysfunction, follow-up duration, sample size, OS, and PFS.

### Risk of bias assessment

The Newcastle-Ottawa scale (NOS) was used to assess the risk of bias in the included studies. The items evaluated included the selection of the study population, comparability between groups, and exposure/outcome assessment. The scale has a total score of 9: a score > 7 indicates high-quality literature, a score of 5–7 indicates moderate-quality literature, and a score ≤ 4 indicates low-quality studies, which were excluded from the analysis.

### Data analysis

Statistical analysis was conducted using the ReMan 5.3 software. Hazard ratio (HR) was selected as the primary statistical indicator for evaluating the prognosis outcome. If HR > 1, it indicated a poor prognosis for the exposed group. For studies reporting HR values, HR and 95% confidence interval (CI) were directly extracted, and the natural logarithm of HR (lnHR) and standard error (SE) of lnHR were calculated using Formula 1. For studies that only provided survival curves, data were extracted from the curves using Engauge Digitizer software, and HR and 95% CI were estimated based on the survival curve calculation method proposed by Tierney et al. (33). Formula 1: SE (lnHR) = (ln (UCI) – ln (LCI))/3.92, where UCI is the upper limit of the confidence interval, LCI is the lower limit of the confidence interval, SE is the standard error, and ln denotes the natural logarithm.

Heterogeneity was assessed using the *I*^2^ statistic and Cochran’s Q test. If *I*² < 50% or *P* > 0.1, heterogeneity was considered non-significant, and a fixed-effect model was used for analysis. If *I*² > 50% or *P* < 0.1, heterogeneity was considered significant, and a random-effects model was used. Subgroup analysis was conducted based on the ethnicity of the study participants and the type of thyroid dysfunction. Sensitivity analysis was performed by sequentially excluding each individual study to assess the impact of individual studies on the overall meta-analysis results for lung cancer prognosis. Publication bias was assessed using Egger’s test, Begg’s test, and funnel plots.

## Results

### Studies screening results

**Figure 1:**
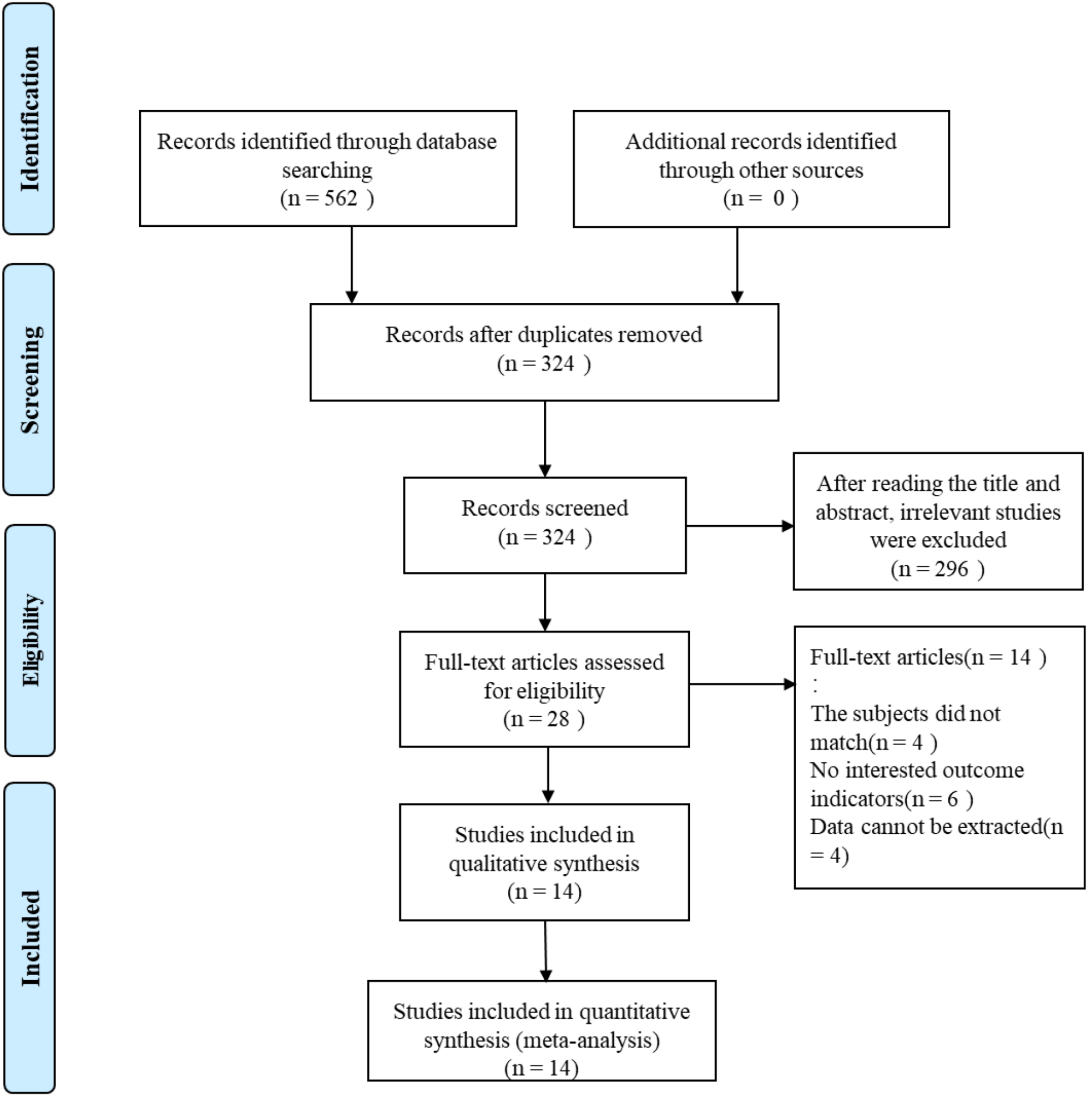
PRISMA flowchart of the studies selection process.

A total of 562 relevant studies were retrieved from the databases. After removing duplicates using EndNote software, 238 duplicate records were excluded, leaving 324 studies for initial screening. By screening the titles and abstracts, the reviewers excluded 296 studies. After carefully reviewing and analyzing the full texts of the remaining 28 studies, 14 studies (24–29, 34–38) involving 2,252 participants were finally included in the meta-analysis (**Figure 1**).

### Basic characteristics of the included studies

The 14 included studies were published between 2017 and 2024. All participants were diagnosed with lung cancer, with most being NSCLC cases and a small number being SCLC patients. The types of immune-related thyroid dysfunction included hypothyroidism and hyperthyroidism. The patients came from seven countries around the world, including France, Italy, Japan, South Korea, China, the United States, and Spain. All patients received anti-PD-1 or anti-PD-L1 treatment. The median or mean follow-up duration ranged from 2.97 months to 33.9 months. The basic characteristics of the included studies are shown in **Table 1**.

**Table 1:** Baseline characteristics of the included studies.

Author, year	Country	Study design	Types of lung cancer	No. of patients	Age (years)	ICI types	Types of thyroid dysfunction	Follow-up Duration
Campredon et al., 2019 (27)	France	Retrospective cohort study	NSCLC	105	61	PD-1 inhibitor	Thyroid dysfunction	9 months
Albertelli et al., 2024 (34)	Italy	Retrospective cohort study	NSCLC	73	65	PD-1 inhibitor	Hypothyroidism	9.1 months
Morimoto et al., 2021 (29)	Japan	Retrospective cohort study	NSCLC	70	69.5	PD-1/PD-L1 inhibitor	Thyroid dysfunction	14.8 months
Kim et al., 2017 (28)	Korea	Retrospective cohort study	NSCLC	58	63.1	PD-1 inhibitor	Thyroid dysfunction	2.97 months
Guo et al., 2024 (26)	China	Retrospective cohort study	NSCLC/SCLC	361	NR	PD-1/PD-L1 inhibitor	Thyroid dysfunction	33.9months
Zhou et al., 2021 (54)	China	Retrospective cohort study	NSCLC	191	58	PD-1 inhibitor	Thyroid dysfunction/hypothyroidism/hyperthyroidism	NR
Osorio et al., 2017 (36)	USA	Prospective cohort study	NSCLC	51	NR	PD-1 inhibitor	Thyroid dysfunction	NR
Wu et al., 2022 (53)	China	Retrospective cohort study	NSCLC/SCLC	29	NR	PD-1 inhibitor	Thyroid dysfunction	31 months
Peiró et al., 2019 (37)	Spain	Retrospective cohort study	NSCLC	71	60.5	PD-1 inhibitor	Thyroid dysfunction	13 months
Yamauchi et al., 2019 (55)	Japan	Retrospective cohort study	Lung cancer	108	NR	PD-1 inhibitor	Thyroid dysfunction	NR
Thuillier et al., 2021 (38)	France	Retrospective cohort study	NSCLC	134	62.5	PD-1 inhibitor	Thyroid dysfunction	10.4 months
Chilelli et al., 2022 (24)	Italy	Retrospective cohort study	NSCLC	75	69	PD-1/PD-L1 inhibitor	Thyroid dysfunction	6.24 months
Luo et al., 2021 (35)	USA	Retrospective cohort study	NSCLC	744	67/63	PD-1 inhibitor	Thyroid dysfunction	18.7/11.1 months
Feng 2022 (25)	China	Retrospective cohort study	NSCLC	182	63.04	PD-1 inhibitor	Thyroid dysfunction/hypothyroidism/hyperthyroidism	NR

NSCLC, non-small-cell lung cancer; SCLC, small-cell lung cancer; ICIs, immune checkpoint inhibitors; PD-1, programmed death receptor 1; PD-L1, programmed cell death-Ligand 1; NR, not reported.

The NOS scores of the included studies ranged from 6 to 9, with two studies scoring 8, nine studies scoring 7, and three studies scoring 6. The overall quality of the included studies was high (**Table 2**).

**Table 2:** Risk of bias assessment.

Author (year)	Selection	Comparability	Outcome/exposure	Total score	Quality
Campredon et al., 2019 (27)	★★★★	★★	★★	8	High
Albertelli et al., 2024 (34)	★★★★	★★	★★	8	High
Morimoto et al., 2021 (29)	★★★	★★	★★	7	Moderate
Kim et al., 2017 (28)	★★★	★★	★★	7	Moderate
Guo et al., 2024 (26)	★★★	★★	★★	7	Moderate
Zhou et al., 2021 (54)	★★★	★★	★★	7	Moderate
Osorio et al., 2017 (36)	★★★	★★	★★	7	Moderate
Wu et al., 2022 (53)	★★	★★	★★	6	Moderate
Peiró et al., 2019 (37)	★★★	★★	★★	7	Moderate
Yamauchi et al., 2019 (55)	★★★	★★	★★	7	Moderate
Thuillier et al., 2021 (38)	★★★	★★	★★	7	Moderate
Chilelli et al., 2022 (24)	★★	★★	★★	6	Moderate
Luo et al., 2021 (35)	★★★	★★	★★	7	Moderate
Feng 2022 (25)	★★	★★	★★	6	Moderate

The asterisk represents the score. One star represents 1 point.

### Meta-analysis

#### Overall survival

**Figure 2:**
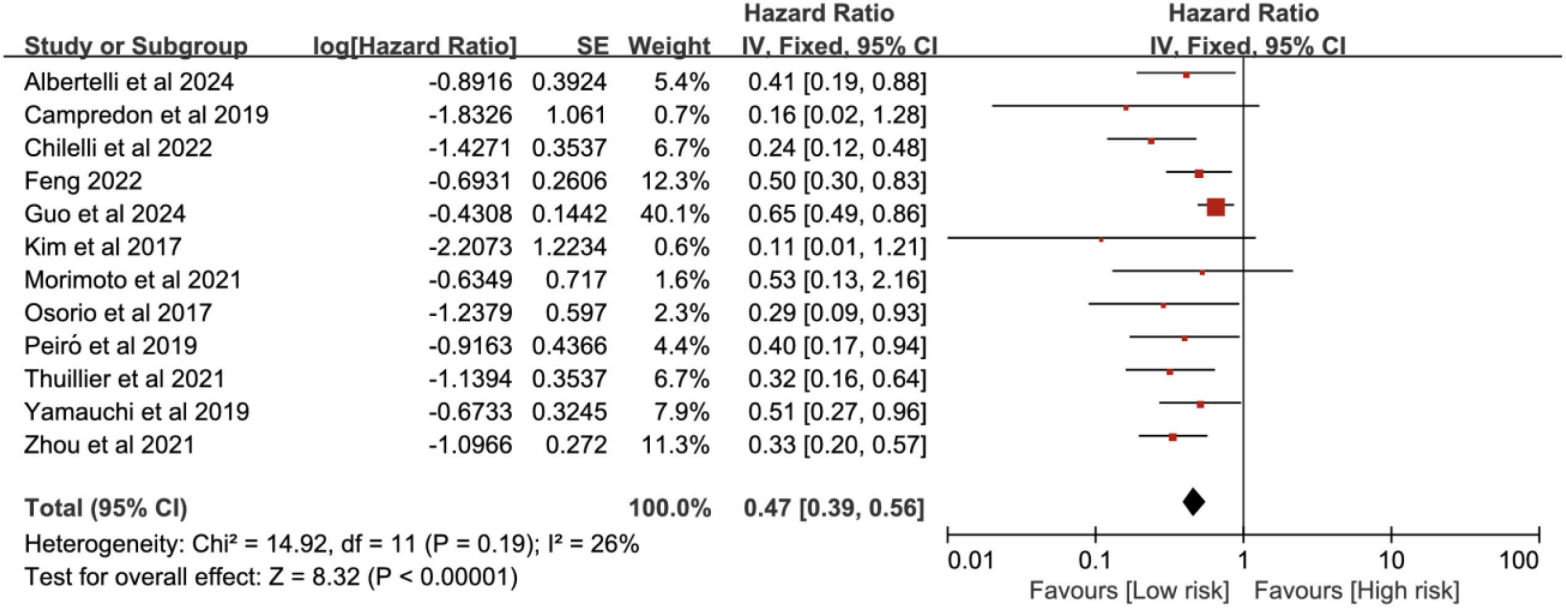
Pooled estimates of the association between immune checkpoint inhibitor-induced thyroid dysfunction and overall survival in lung cancer patients.

A total of 12 studies were included in the OS analysis. Heterogeneity assessment results showed *P =* 0.19, *I*^2^ = 26%, indicating no significant heterogeneity. The analysis was conducted using the fixed-effect model. The meta-analysis demonstrated that ICI-induced immune-related thyroid dysfunction was associated with improved OS in patients with lung cancer (HR = 0.47, 95% CI [0.39, 0.56], *P* < 0.00001) (**Figure 2**).

#### Progression-free survival

**Figure 3:**
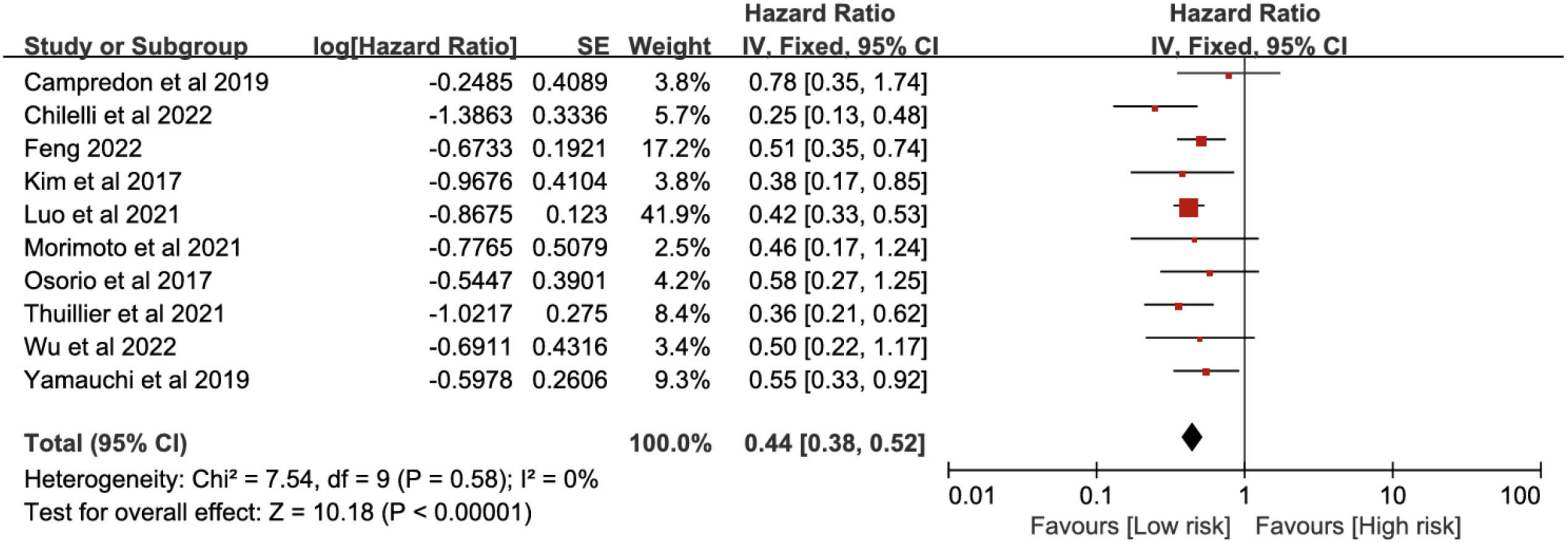
Pooled estimates of the association between immune checkpoint inhibitor-induced thyroid dysfunction and progression-free survival in lung cancer patients.

A total of 10 studies were included in the PFS analysis. Heterogeneity test results showed *P* = 0.58, *I*^2^ = 0%, indicating no significant heterogeneity. A fixed-effect model was used for analysis. The meta-analysis results showed that ICI-induced immune-related thyroid dysfunction significantly improved the PFS of patients with lung cancer (HR = 0.44, 95% CI [0.38, 0.52], *P* < 0.00001) (**Figure 3**).

### Subgroup analysis

**Figure 4:**
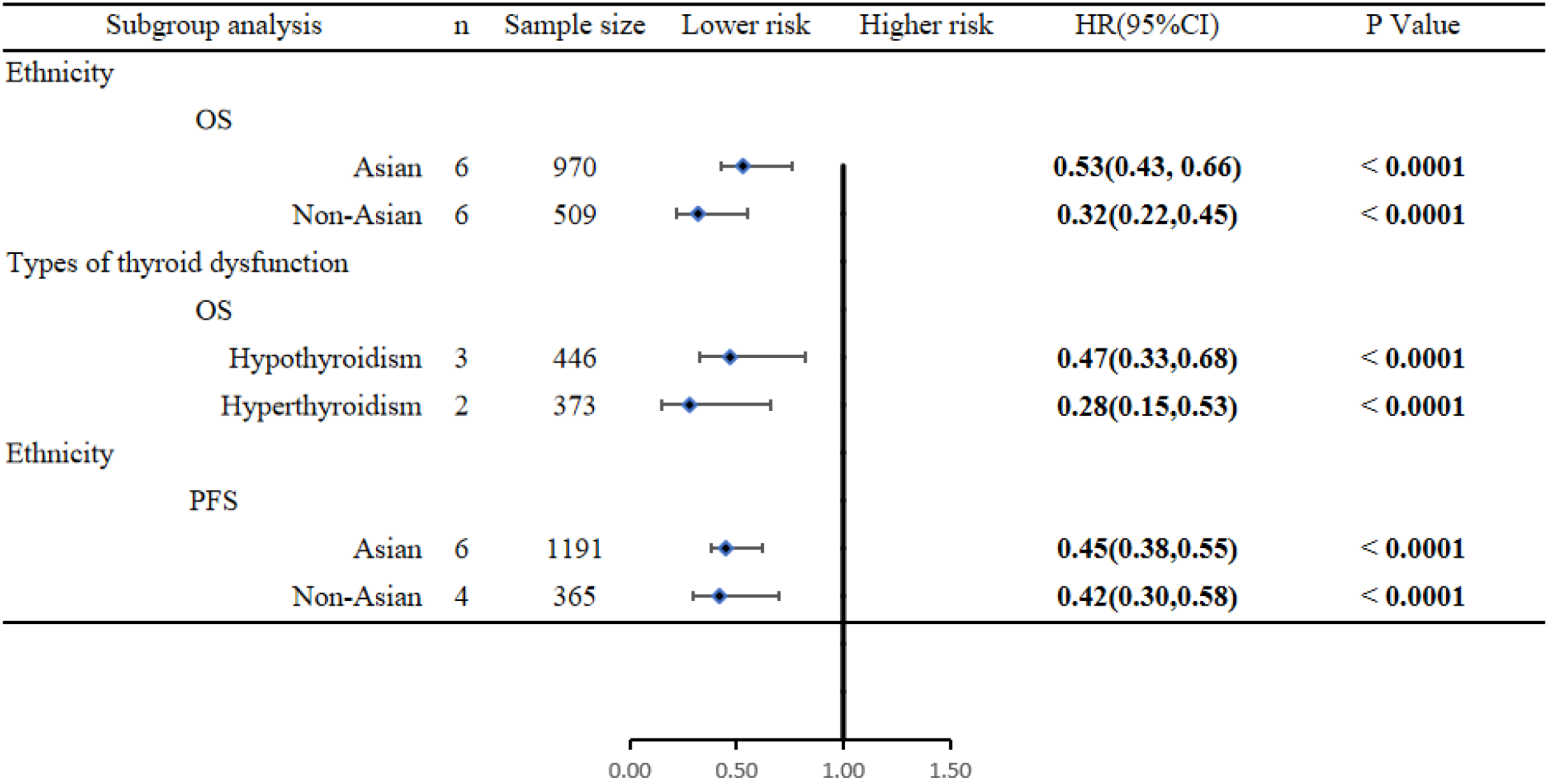
Subgroup analysis results.

Subgroup analysis based on the ethnicity of the study participants showed that in both Asian and non-Asian populations, ICI-induced immune-related thyroid dysfunction improved OS (Asian: HR = 0.53, 95% CI [0.43, 0.66], *P* < 0.00001; Non-Asian: HR = 0.32, 95% CI [0.22, 0.45], *P* < 0.00001) and PFS of patients with lung cancer (Asian: HR = 0.45, 95% CI [0.38, 0.55], *P* < 0.00001; non-Asian: HR = 0.42, 95% CI [0.30, 0.58], *P* < 0.00001) (**Figure 4**).

Subgroup analyses were performed based on the type of thyroid dysfunction. The subgroup analysis revealed that ICI-induced hypothyroidism and hyperthyroidism could improve the OS of patients with lung cancer (hypothyroidism: HR = 0.47, 95% CI [0.33, 0.68], *P* < 0.00001; hyperthyroidism: HR = 0.28, 95% CI [0.15, 0.53], *P* < 0.00001) (**Figure 4**).

### Sensitivity analysis

**Figure 5:**
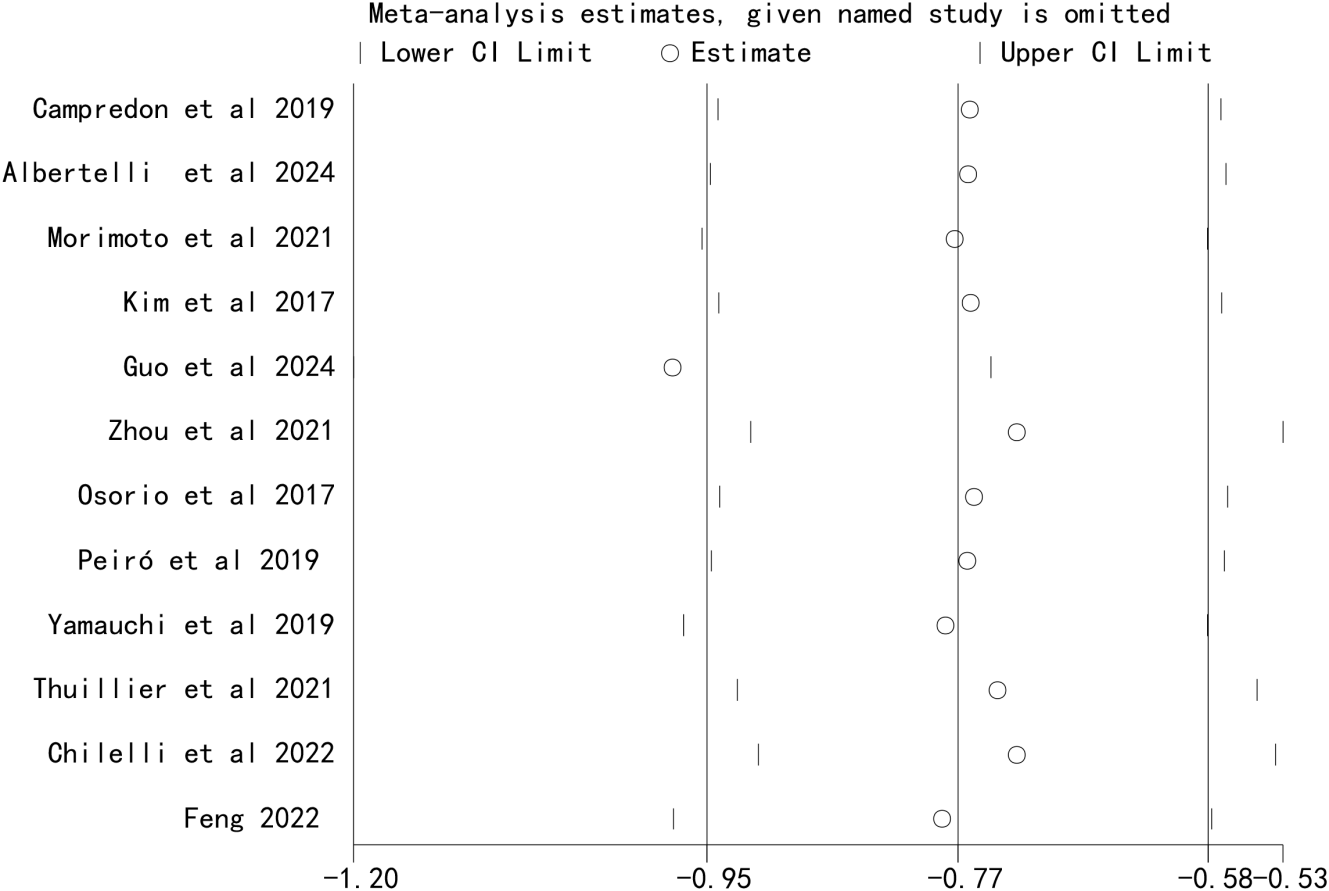
Sensitivity analysis for the association between immune checkpoint inhibitor-induced thyroid dysfunction and overall survival in lung cancer patients.

**Figure 6:**
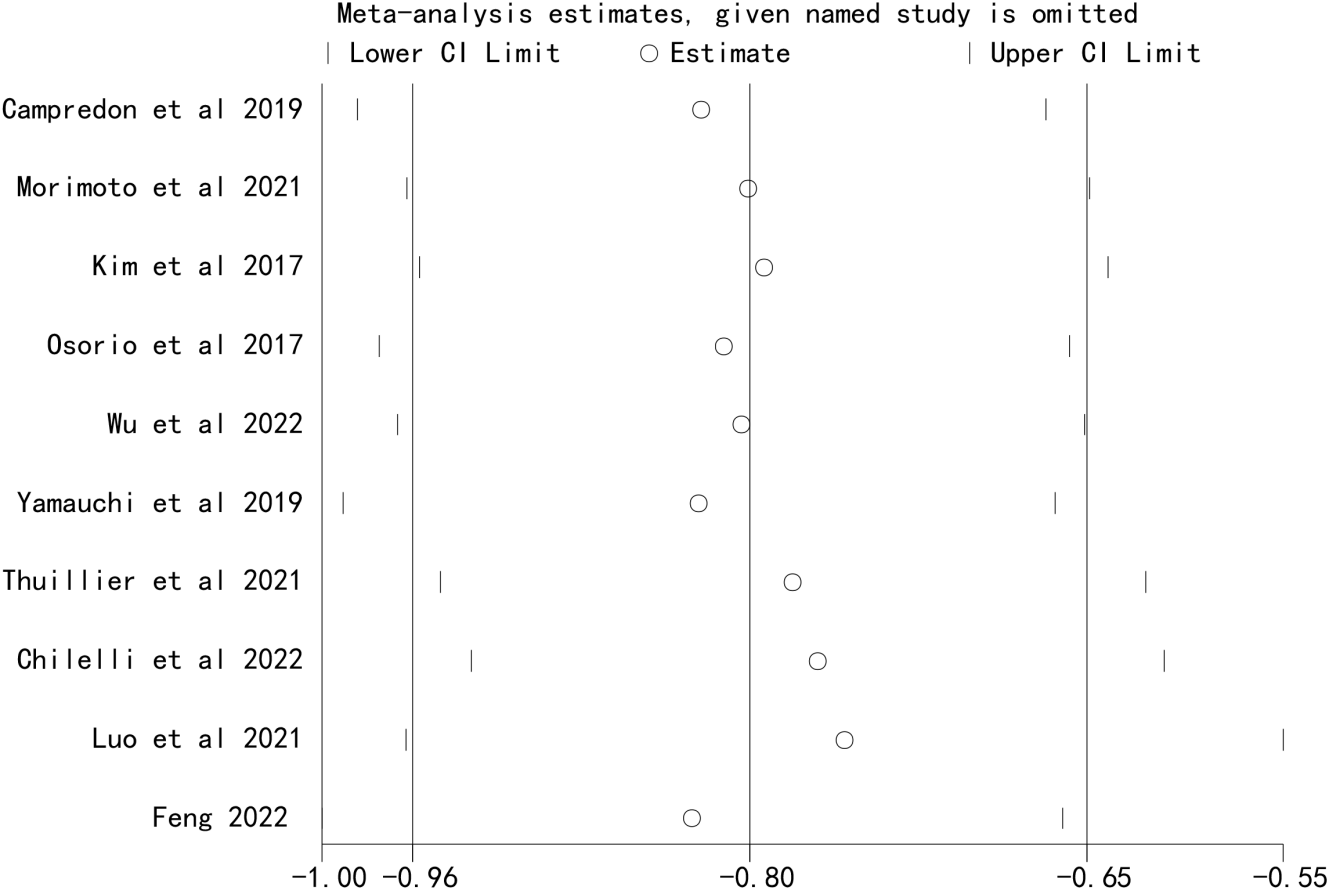
Sensitivity analysis for the association between immune checkpoint inhibitor-induced thyroid dysfunction and progression-free survival in lung cancer patients.

Sensitivity analyses were performed by sequentially excluding each included study. The results showed that the pooled effect size of the association between ICI-induced immune-related thyroid dysfunction and the prognosis of patients with lung cancer remained stable, indicating that the results of this meta-analysis were robust (**Figures 5**, **6**).

### Publication bias

**Figure 7:**
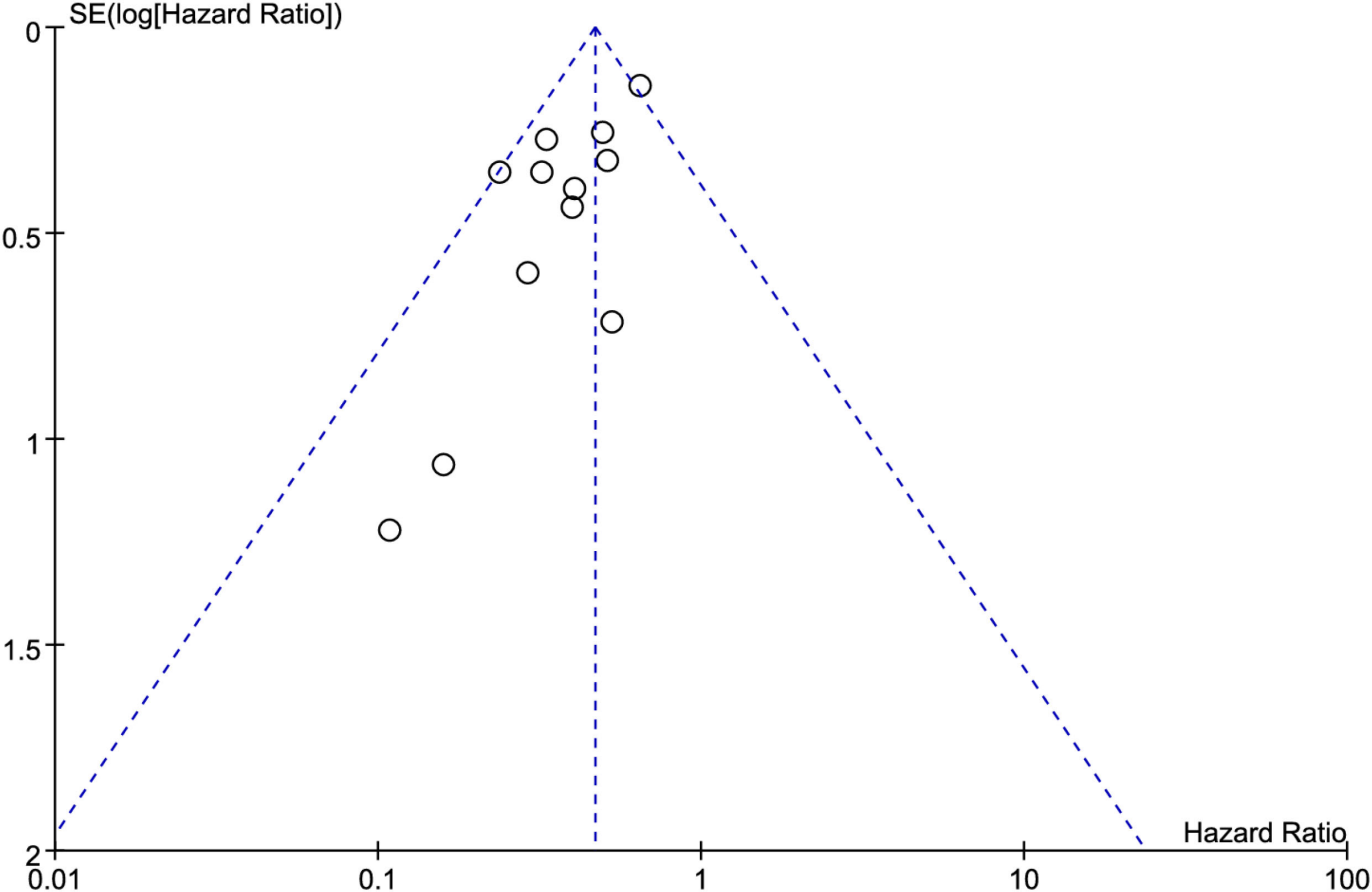
Funnel plots for the association between immune checkpoint inhibitor-induced thyroid dysfunction and overall survival in lung cancer patients.

**Figure 8:**
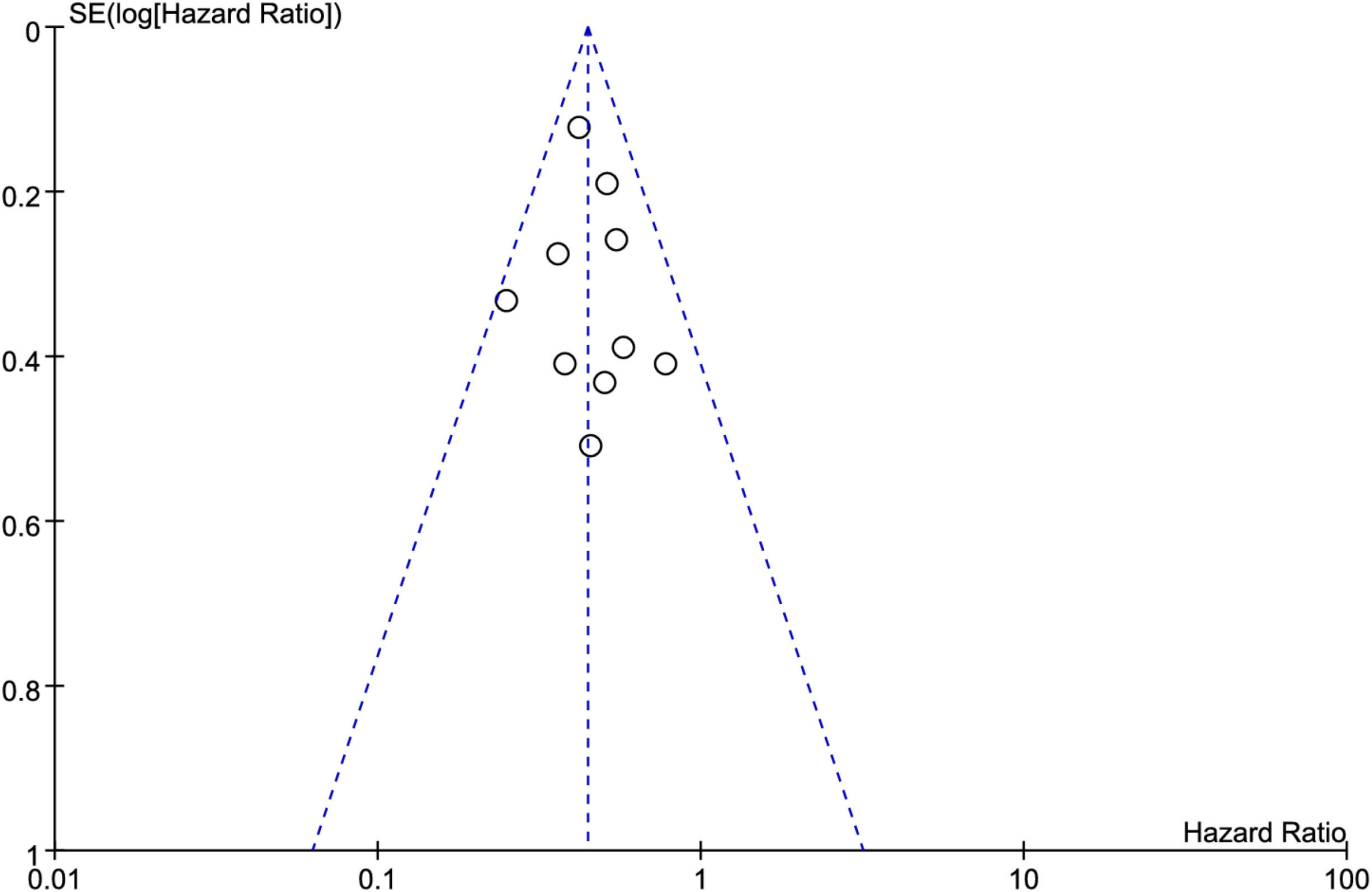
Funnel plots for the association between immune checkpoint inhibitor-induced thyroid dysfunction and progression-free survival in lung cancer patients.

Funnel plots were used to visually assess publication bias. The results showed that the points on both sides of the funnel plot were generally symmetrical, indicating no publication bias (**Figures 7**, **8**). In addition, Egger’s test and Begg’s test also showed no significant publication bias (**Table 3**).

**Table 3:** Publication bias analysis.

Outcome	Egger’s tests (p-value)	Begg’s tests (p-value)
Overall survival	0.219	0.056
Progression-free survival	0.325	0.082

## Discussion

Through a comprehensive literature review and rigorous meta-analysis, this study clarified the association between immune-related thyroid dysfunction and the prognosis of patients with lung cancer. The results showed that ICI-induced immune-related thyroid dysfunction could significantly improve the OS and PFS of patients with lung cancer, which has important clinical significance and potential biological mechanisms.

A study by Kim et al. (28) demonstrated that among NSCLC patients treated with ICIs, those with thyroid dysfunction had significantly better OS and PFS than those without thyroid dysfunction. A study by Lima Ferreira et al. (39) indicated that the OS of patients with abnormal thyroid function was 3.27 years, while that of patients without abnormal thyroid function was 1.76 years. This further supports the idea that abnormal thyroid function may be associated with a better treatment response. A relevant meta-analysis (40, 41) found that the development of immune-related thyroid diseases was associated with the improvement of OS and PFS during ICI treatment. Despite potential heterogeneity and biases, the evidence still indicates that thyroid dysfunction can serve as a potential surrogate marker for immune treatment response.

Over the past decade, immunotherapy has fundamentally changed the treatment landscape for solid tumors and hematological malignancies. Although most irAEs are self-limiting, they may affect the course and efficacy of immunotherapy, as well as patients’ OS. Among these, immune-related thyroid dysfunction is the most common endocrine-related adverse event. To date, the pathogenesis of ICI-related thyroid dysfunction has not been fully elucidated, but its core lies in the synergistic effect of ICI-mediated immune tolerance imbalance and thyroid tissue-specific susceptibility (42–44). The core hypothesis is that ICIs disrupt the body’s immune tolerance by blocking immune checkpoint pathways, activating autoreactive T cells that specifically target thyroid tissue, accompanied by the production of autoantibodies, cytokine storms, and other immune abnormalities, ultimately resulting in the destruction of thyroid follicular structure and functional impairment (44, 45). Specifically, this process involves several key steps: activation and proliferation of autoreactive T cells induced by immune checkpoint pathway blockade, specific susceptibility of thyroid tissue, thyroid tissue damage caused by immune cell infiltration and cytokine storms, involvement of autoantibodies, and regulation by genetic susceptibility factors.

The PD-1/PD-L1 pathway is a key mechanism for maintaining peripheral immune tolerance in thyroid tissue. Anti-PD-1/PD-L1 antibodies block this binding process, relieving the inhibition of autoreactive T cells, which then become continuously activated and proliferate, acquiring potent cytotoxic activity to specifically recognize and target thyroid follicular epithelial cells (46, 47). Yamauchi et al. (48) found that the mRNA levels of PD-L1 and PD-L2 in normal thyroid tissue are highly expressed, which are significantly higher than those. This finding suggests that thyroid tissue may maintain autoimmune tolerance by highly expressing PD-L1/PD-L2 to bind to PD-1 on immune cells; however, when ICIs block the PD-1/PD-L1 pathway, this ‘protective’ mechanism is disrupted, rendering thyroid tissue a preferential target for activated T cells. Specific antigens expressed by thyroid tissue (e.g., thyroid peroxidase, thyroglobulin, and thyroid-stimulating hormone receptor) may also be involved in the specific recognition process during immune attack. After autoreactive T cells activated by ICIs infiltrate thyroid tissue, they trigger local inflammatory responses, accompanied by the recruitment and activation of various immune cells and the formation of a cytokine storm, which ultimately results in the destruction of thyroid follicular structure and impaired thyroid function (44, 47).

CTLA-4 gene polymorphisms may impair the body’s immune tolerance by affecting the expression or function of CTLA-4 protein, increasing the risk of autoimmune thyroid diseases (49, 50). HLA gene polymorphisms may also be involved in the development of ICI-related thyroid dysfunction (51, 52). However, key scientific questions—such as why thyroid tissue becomes a preferential target for ICI-induced immune attack, differences in the pathogenesis of different types of ICIs, and the specific regulatory role of genetic factors—remain to be further clarified.

Regarding ethnicity, both Asian and non-Asian populations with immune-related thyroid dysfunction showed a significant improvement in OS and PFS, but there were differences in the magnitude of the effect: immune-related thyroid dysfunction appeared to have a more pronounced impact on OS and PFS improvement in non-Asian populations. Regarding the type of immune-related thyroid dysfunction, both hypothyroidism and hyperthyroidism significantly prolonged OS in patients with lung cancer, with hyperthyroidism exerting a more pronounced effect on OS improvement. These findings not only provide strong evidence for the importance of thyroid function monitoring during immunotherapy in patients with lung cancer but also lay a theoretical foundation for clinicians to develop more precise treatment strategies based on patients’ ethnicity and type of thyroid dysfunction. The results of this study have important guiding significance for the development and monitoring of immunotherapy regimens for patients with lung cancer and can provide a strong basis for clinical decision-making.

In terms of treatment regimen development, for patients with lung cancer receiving ICI treatment, doctors should attach great importance to the monitoring of thyroid function. On the one hand, the association between thyroid dysfunction and better prognosis supports its potential as a clinical biomarker for predicting ICI efficacy, helping to identify patient populations who may derive greater benefit from immunotherapy at an early stage. On the other hand, it is crucial to emphasize that this association does not mean that the standardized management of thyroid dysfunction itself can be ignored. Untreated hypothyroidism or hyperthyroidism can significantly impair patients’ quality of life and even lead to serious complications such as cardiovascular events. Therefore, clinical practice should strictly follow guidelines, regularly monitor thyroid function, and provide standardized treatment for confirmed thyroid dysfunction.

### Limitations

This study has several limitations. First, although 2,252 patients with lung cancer were included, the sample size is still relatively small compared with the total number of patients with lung cancer worldwide. Second, the included studies were mainly from Europe, North America, and Asia, so the generalizability of the results to patients with lung cancer in other regions may be limited. Third, most of the included studies were retrospective observational studies, which cannot establish a causal relationship and are prone to selection bias, information bias, and residual confounding. These biases may overestimate the strength of the association between thyroid dysfunction and improved prognosis. Fourth, although heterogeneity assessment showed no significant statistical heterogeneity among studies, other potential sources of heterogeneity cannot be completely ruled out. However, the consistent direction of effect across most analyses supports the robustness of the meta-analysis results. Fifth, Egger’s test, Begg’s test, and funnel plots showed no publication bias; however, since this meta-analysis only included Chinese and English studies, language bias may exist. Finally, due to the limited data provided by the included studies, we were only able to perform subgroup analyses based on ethnicity and type of thyroid dysfunction and could not analyze other potential confounding factors. Future studies should further expand the sample size and include patients with lung cancer from diverse regions and ethnicities to enhance the generalizability and representativeness of the results.

## Conclusion

In summary, our analysis shows that patients with lung cancer who develop thyroid dysfunction (either hypothyroidism or hyperthyroidism) during immunotherapy may have longer OS and prolonged tumor control duration. This suggests that for clinicians, monitoring thyroid function is not only necessary for managing treatment-related adverse events but may also provide a valuable clue to assess treatment efficacy. Future studies should explore the potential of immune-related thyroid dysfunction as a biomarker for predicting the efficacy and prognosis of immunotherapy in patients with lung cancer.

## Data Availability

The datasets presented in this study can be found in online repositories. The names of the repository/repositories and accession number(s) can be found in the article/**Supplementary Material**.
