# Supplementary material for: Immune checkpoint inhibitors-induced thyroid dysfunction improves the prognosis of patients with lung cancer: a meta-analysis and systematic review
Source: Front Endocrinol (Lausanne). 2026 Jan 20;16:1743245. doi: 10.3389/fendo.2025.1743245 (PMC12864112; doi:10.3389/fendo.2025.1743245)
Supplement: Supplementary file 2 [file Table2.docx]

| **Supplementary Materials Table S2.Search strategy in PubMed** | |
| --- | --- |
| #1 | (Immune Checkpoint Inhibitors[MeSH Terms]) OR (Checkpoint Inhibitors, Immune[tiab]) OR (Immune Checkpoint Inhibitor[tiab]) OR (Checkpoint Inhibitor, Immune[tiab]) OR (Immune Checkpoint Blockers[tiab]) OR (Checkpoint Blockers, Immune[tiab]) OR (Immune Checkpoint Blockade[tiab]) OR (Checkpoint Blockade, Immune[tiab]) OR (Immune Checkpoint Inhibition[tiab]) OR (Checkpoint Inhibition, Immune[tiab]) OR (PD-L1 Inhibitors[tiab]) OR (PD L1 Inhibitors[tiab]) OR (PD-L1 Inhibitor[tiab]) OR (PD L1 Inhibitor[tiab]) OR (Programmed Death-Ligand 1 Inhibitors[tiab]) OR (Programmed Death Ligand 1 Inhibitors[tiab]) OR (PD-1-PD-L1 Blockade[tiab]) OR (Blockade, PD-1-PD-L1[tiab]) OR (PD 1 PD L1 Blockade[tiab]) OR (CTLA-4 Inhibitors[tiab]) OR (CTLA 4 Inhibitors[tiab]) OR (CTLA-4 Inhibitor[tiab]) OR (CTLA 4 Inhibitor[tiab]) OR (Cytotoxic T-Lymphocyte-Associated Protein 4 Inhibitors[tiab]) OR (Cytotoxic T Lymphocyte Associated Protein 4 Inhibitors[tiab]) OR (Cytotoxic T-Lymphocyte-Associated Protein 4 Inhibitor[tiab]) OR (Cytotoxic T Lymphocyte Associated Protein 4 Inhibitor[tiab]) OR (PD-1 Inhibitors[tiab]) OR (PD 1 Inhibitors[tiab]) OR (PD-1 Inhibitor[tiab]) OR (Inhibitor, PD-1[tiab]) OR (PD 1 Inhibitor[tiab]) OR (Programmed Cell Death Protein 1 Inhibitor[tiab]) OR (Programmed Cell Death Protein 1 Inhibitors[tiab]) OR (nivolumab[tiab]) OR (pembrolizumab[tiab]) OR (atezolizumab[tiab])) OR (ipilimumab[tiab]) OR (camrelizumab[tiab]) OR (toripalimab[tiab]) OR (tislelizumab[tiab]) OR (sintilimab[tiab]) OR (durvalumab[tiab]) OR (envafolimab[tiab]) OR (cemiplimab[tiab]) OR (oleclumab[tiab]) |
| #2 | ("Thyroid Diseases"[Mesh] OR "Hypothyroidism"[Mesh] OR "Hyperthyroidism"[Mesh] OR"Thyroiditis"[Mesh] OR "Immune System Diseases"[Mesh]) OR(("thyroid dysfunction"[tiab] OR "thyroid disorder*"[tiab] OR "thyroid adverse event*"[tiab]) OR ("hypothyroid*"[tiab]) OR("hyperthyroid*"[tiab] OR "thyrotoxicosis"[tiab]) OR ("thyroiditis"[tiab] OR "silent thyroiditis"[tiab]) OR ("immune-related adverse event*"[tiab] OR irAE*[tiab])) |
| #3 | ("Lung Neoplasms"[Mesh] OR "Carcinoma, Non-Small-Cell Lung"[Mesh] OR "Small Cell Lung Carcinoma"[Mesh]) OR (("lung cancer"[tiab] OR "lung cancers"[tiab] OR "lung neoplasm*"[tiab] OR "lung tumor*"[tiab] OR "lung tumour*"[tiab] OR "pulmonary cancer"[tiab] OR "pulmonary neoplasm*"[tiab]) OR ("non-small cell lung cancer"[tiab] OR NSCLC[tiab] OR "lung adenocarcinoma"[tiab] OR "lung squamous cell carcinoma"[tiab]) OR ("small cell lung cancer"[tiab] OR SCLC[tiab])) |
| #4 | ("Prognosis"[Mesh] OR "Survival Analysis"[Mesh] OR "Disease-Free Survival"[Mesh] OR "Overall Survival"[Mesh] OR "Progression-Free Survival"[Mesh]) OR ((prognos*[tiab] OR outcome*[tiab] OR surviv*[tiab]) OR ("overall survival"[tiab] OR OS[tiab]) OR ("progression free survival"[tiab] OR "progression-free survival"[tiab] OR PFS[tiab]) OR ("disease free survival"[tiab] OR "disease-free survival"[tiab] OR DFS[tiab])) |
| #5 | #1 AND #2 AND #3 AND #4 |
